# The Antipsychotic Thioridazine Shows Promising Therapeutic Activity in a Mouse Model of Multidrug-Resistant Tuberculosis

**DOI:** 10.1371/journal.pone.0012640

**Published:** 2010-09-09

**Authors:** Dick van Soolingen, Rogelio Hernandez-Pando, Hector Orozco, Diana Aguilar, Cecile Magis-Escurra, Leonard Amaral, Jakko van Ingen, Martin J. Boeree

**Affiliations:** 1 National Mycobacteria Reference Laboratory, Laboratories for Infectious Diseases and Perinatal Screening, National Institute for Public Health and the Environment, Bilthoven, The Netherlands; 2 Departments of Clinical Microbiology and Respiratory Diseases, Radboud University Nijmegen Medical Centre, Nijmegen, The Netherlands; 3 Experimental Pathology Section, Department of Pathology, National Institute of Medical Sciences and Nutrition Salvador Zubiràn, Mexico City, Mexico; 4 University Centre for Chronic Diseases Dekkerswald, Radboud University Nijmegen Medical Centre, Nijmegen, The Netherlands; 5 Mycobacteriology Unit, Institute of Hygiene and Tropical Medicine, Universidade Nova de Lisboa, Lisbon, Portugal; University of Cape Town, South Africa

## Abstract

Multidrug- and extensively drug-resistant tuberculosis have emerged as grave threats to public health worldwide. Very few active drugs are available or likely to become available soon. To address these problems we revisited a classical observation, the applicability of phenothiazines as antimicrobial drugs. Within this pharmacological class we selected thioridazine, which is most efficacious and least toxic, when used as an antipsychotic drug. We tested thioridazine monotherapy in the Balb/c mouse model for its activity to treat both susceptible and multidrug-resistant tuberculosis by a two months daily oral administration of 32 and 70 mg/kg. In addition, we tested its additive value when combined with a standard first-line regimen for susceptible tuberculosis. Thioridazine treatment resulted in a significant reduction of colony-forming-units of the susceptible (−4.4 log CFU, p<0.05) and multidrug-resistant tuberculosis bacilli (−2.4 log CFU, p<0.009) in the lung both at one and two months after infection, compared to controls. Moreover, when thioridazine was added to a regimen containing rifampicin, isoniazid and pyrazinamide for susceptible tuberculosis, a significant synergistic effect was achieved (−6.2 vs −5.9 log CFU, p<0.01). Thioridazine may represent an effective compound for treatment of susceptible and multidrug-resistant tuberculosis. The phenothiazines and their targets represent interesting novel opportunities in the search for antituberculosis drugs.

## Introduction

The phenothiazines are a class of antipsychotic drugs that have activity against the plasma membrane of the responsive eukaryotic cells and, hence, also against similar membranes of prokaryotic cells. The broad spectrum antimicrobial activity of chlorpromazine, the first commercially available phenothiazine, was first recognized in the 1950s; its activity was thought to result from binding to penicillin binding proteins, thereby inhibiting cell wall synthesis [1]. The serious side effects, including hepatotoxicity and agranulocytosis, associated with chronic use of chlorpromazine for psychoses limited the interest in its antimicrobial activity.

In the following decades less toxic phenothiazine derivates, including thioridazine, were developed. They also proved to have a potent in vitro activity against a variety of bacteria. However, many effective antibiotics became available during the so-called golden era of antibiotics and phenothiazines were thus no longer considered in the treatment of bacterial infections [2].

The activity of the phenothiazines against mycobacteria has gained renewed attention due to the ongoing spread of multidrug-resistant tuberculosis (MDR-TB), i.e. tuberculosis caused by *Mycobacterium tuberculosis* complex bacteria that are resistant to at least isoniazid and rifampicin [2]. These two drugs are the most potent drugs used in TB treatment. Worldwide, on average 5.3% of the TB cases can be classified as MDR-TB, according to the WHO report of March 2008 [3]. The emergence of MDR-TB strains that reveal additional resistance against fluoroquinolones and aminoglycosides (extensively drug-resistant TB, or XDR-TB) marks a new point in the history of the long-lasting battle against TB [3].

New drugs are in the pipeline, including the diarylquinoline TMC207 which was recently evaluated in a phase II study [4], [5]. Still, it will take years before their efficacy and safety have been evaluated and before they become available in resource-poor settings where the burden of resistance is largest and untreatable TB cases emerge rapidly [4], [5].

The *in vitro* antituberculosis activity of thioridazine has been well established [6], [7]. In addition, exposure of immune cells to thioridazine has been shown to promote the release of cytokines involved in the mounting of a response to mycobacteria [6]. These cytokines are also markers of progression of a tuberculosis infection to active disease status [8]. Thioridazine has the additional advantage that it has been registered for a prolonged period of time and has become relatively inexpensive. Moreover, thioridazine has been used extensively as an antipsychotic drug, generally without causing severe side effects [2].

Given the emergence of resistant forms of tuberculosis and the favourable antimicrobial and immunomodulatory characteristics of thioridazine, we revisited a classical observation, the applicability of phenothiazines in the treatment of bacterial infections.

## Methods

### Ethics Statement

All animal work was performed in conformity with the local Ethical Committee for Experimentation in Animals in Mexico; these are laid out in document NOM 062-ZOO-1999: Technical rules for the production, care and use of laboratory animals. The study was approved by the institutional review board at the National Institute of Medical Sciences and Nutrition Salvador Zubiràn under file number PAT-037-06-09-1.

### Experimental model of tuberculosis infection in mice

The tuberculosis Balb/c mouse model has been described in detail previously [9]. In short: Male Balb/c mice were obtained from Jackson Laboratories (Bar Harbor, Maine, USA) and used at 6–8 weeks of age. Virulent *M. tuberculosis* H37Rv and a MDR clinical isolate (code 900) isolated from a tuberculosis patient in Monterrey city, northern Mexico, were cultured in Youman's modification of the Proskauer/Beck medium. The MDR strain, which belongs to the X1 genotype based on spoligotyping, was resistant to rifampicin (MIC 100 µg/ml), isoniazid (MIC 3.13 µg/ml), ethambutol (MIC 8 µg/ml) and streptomycin (MIC>100 µg/ml); its rifampicin resistance resulted from a Ser531Leu mutation in the *rpoB* gene. Colonies were harvested after four weeks and suspended in phosphate-buffered saline (PBS) containing 0.05% Tween 80 and shaken for 10 min together with glass beads. The suspension was centrifuged for 1 min at 350×g to sediment large clumps of bacilli. Thereafter, a preliminary microscopic bacterial count was conducted by analyzing the supernatant at a known ratio of volume to area, and counting 10 random fields after staining by the Ziehl-Neelsen staining technique. The suspension was adjusted to 2.5×10^5^ bacteria in 100 µl of PBS and stored at −70°C. Before using the bacteria taken from the freezer stock, they were recounted, and the viability was checked as previously described [10].

To achieve intratracheal infection, mice were anaesthetized by intraperitoneal application of 56 mg/kg thiopental. Thereafter, the trachea was exposed via a small midline incision, by injection of 2.5×10^5^ viable bacteria in 100 µl of PBS. The incision was sutured with sterile silk, and the mice were maintained vertically until the effects of the anaesthetic had worn off. Infected animals were housed in groups of five in cages fitted within micro-isolators.

### Drug dosing and administration

First, a dose finding study was undertaken in mice infected with H37Rv. The selection of the appropriate thioridazine dose was done following previous publications [11]; we tested 16, 32 and 70 mg/kg dosages. Treatment was started 60 days after infection, when tuberculosis was already well advanced. Surviving animals were randomly allocated to the three treatment groups of 20 mice each. The thioridazine dosages were administered daily as dissolved drug in distilled water in a total volume of 0.1 ml administered daily by an intragastric cannula; treatment duration was 2 months.

In a second experiment, we repeated infection and treatment using the clinical MDR-TB isolate. Based on findings in experiment one, only 32 mg/kg and 70 mg/kg of thioridazine were used.

In the third experiment 60 mice were infected with H37Rv. Mice surviving 60 days after infection were randomly assigned to three experimental groups. The first group of 20 infected mice was treated by conventional chemotherapy: rifampicin (10 mg/kg), isoniazid (10 mg/kg), and pyrazinamide (30 mg/kg), administered daily by an intragastric cannula, plus 32 mg/kg of thioridazine administered daily by the same route in separate solutions. The second group of 20 infected mice exclusively received the conventional chemotherapy, and the control group was solely treated with the diluent. Again, treatment duration was 2 months.

All three experiments were done in duplicate. In all experiments, four mice per group were sacrificed by exsanguination under terminal anaesthesia after 2, 4 and 8 weeks of treatment; no animals were sacrificed at the start of treatment. The bacillary load of the lungs was determined by colony forming units (CFU) quantification and observation of the histological damage as described below.

In all experiments, a subset of animals from each group was sacrificed at one and two month intervals. All data points are means (±SD) of results in four animals and represent results in two separate experiments that yielded highly reproducible results.

### Bacteriological and histological assessment of infected lungs

During dissection of the animals, one lung lobe (right or left) was immediately frozen by immersion in liquid nitrogen and used for colony counting, while the other was perfused with 10% formaldehyde and used for histopathology analysis. For CFU determination, frozen lungs were disrupted in a Polytron homogeniser (Kinematica, Luzern, Switzerland) in sterile 50 mL tubes containing 3 ml of isotonic saline. Four dilutions of each homogenate were spread onto duplicate Middlebrook 7H10 agar plates enriched with OADC; plates were incubated for 21 days. Four animals were sacrificed at each time point and all experiments were performed in duplicate; hence, the data points are the means of eight animals.

For the histological study, after two days of fixation, parasaggital sections were taken through the hilus from 4 lung lobes per time point in two separate experiments, and these were dehydrated and embedded in paraffin, sectioned at 5 µM and stained with haematoxylin and eosin. The percentage of lung affected by pneumonia was measured using a Zidas Zeiss image analysis system. Measurements were done in a blinded manner, by a certified pathologist, and data are expressed as the mean of 8 animals ±SD.

### Statistics

A one way analysis of variance (ANOVA) and Student's t-test were used to compare numbers of CFUs and morphometry determinations in infected mice treated with thioridazine and non-treated control animals. A difference of p<0.05 was considered statistically significant.

## Results

**Figure 1:**
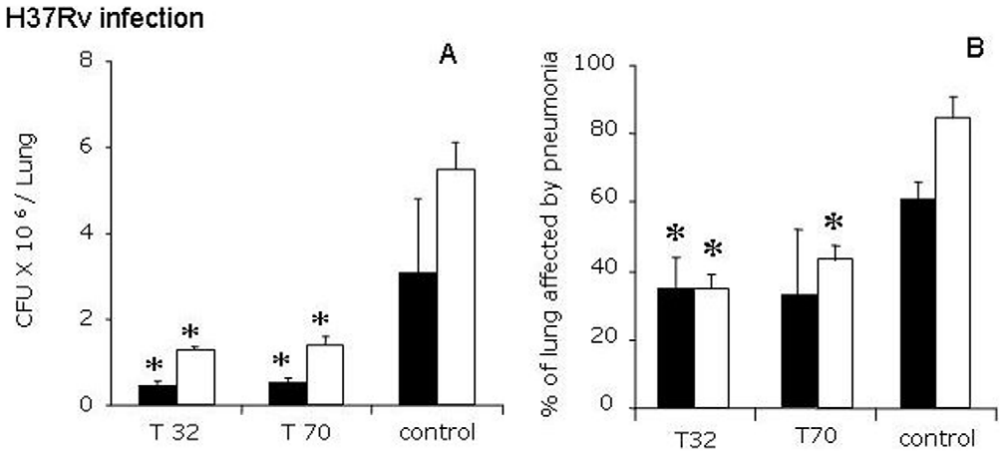
Effect of Thioridazine administration on the bacillary loads (CFU) and histological damage of mice infected with drug susceptible strain H37Rv strain. (A) Both doses of 32 (T32) and 70 (T70) mg/kg of thioridazine administered daily from day 60 after infection significantly reduced CFU by day 30 (black bars) and 60 (white bars) after initiation of treatment. CFU rose progressively in the untreated control animals. (B) In the thioridazine treated animals, the percentage of the lung surface affected by pneumonia was significantly smaller in thioridazine treated- than in control animals after one (black bars) and two months (white bars) of treatment. Data is expressed as means ± SD, 8 mice per time point, asterisks represent statistical significance (p<0.05).

In the first experiment, in animals infected with the drug-susceptible *M. tuberculosis* reference strain H37Rv, the lowest dose of thioridazine (16 mg/kg) was almost ineffective as this did not result in a clear reduction of the number of bacilli in the lung (data not shown), while the intermediate (32 mg/kg) and high dose (70 mg/kg) yielded a significant killing of H37Rv bacilli (Figure 1). As shown in Figure 1, after 30 days of treatment a significant four fold decrease in the lung bacillary loads was found in the thioridazine-treated mice (6×10^5^, SD 1×10^5^), compared to the control group (2.7×10^6^ SD 1.5×10^6^, p<0.019). Thereafter, a slight rise in the number of CFU was observed in both treated groups by day 60. However, a five fold decrease was seen in the treated animals (1.1×10^6^, SD 5×10^4^) when compared to the control group (5.5×10^6^, SD 6×10^5^, p<0.05). Latter group showed a progressive increase in CFUs in the lung (Figure 1). These results on bacillary loads correlate well with the morphometric observations, which showed a significant decrease of the lung area affected by pneumonia in the treated animals when compared to the non-treated control group (Figure 1B).

**Figure 2:**
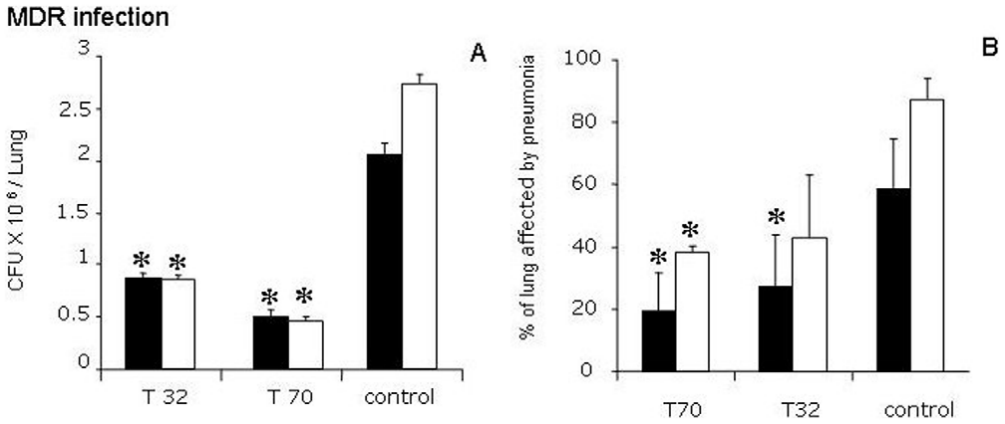
Effect of Thioridazine administration on the bacillary loads (CFU) and histological lung damage of mice infected with the MDR-TB strain. (A) In comparison with the none-treated control group of mice, both doses of thioridazine significantly reduced the CFU, 30 (black bars) and 60 (white bars) days after initiation of treatment. CFU rose progressively in the untreated control animals. (B) In the thioridazine treated animals, the percentage of the lung surface affected by pneumonia was found significantly lower than in control animals after one (black bars) and two months (white bars) of treatment. Data is expressed as means ± SD, 8 mice per time point, asterisks represent statistical significance (p<0.05).

In the second experiment we observed that the dose of 70 mg/kg of thioridazine, daily administered in mice infected with an MDR-TB strain significantly reduced the CFUs at 30 and 60 days (5.1×10^5^, SD 6×10^4^) after initiation of treatment (p<0.009), whereas CFUs rose progressively in the untreated control animals (2.75×10^6^, SD 5×10^4^. Figure 2). A lesser, but still significant decrease of the pulmonary bacillary load was observed in MDR-TB infected animals treated with 32 mg/kg of thioridazine (p<0.025). In agreement with this observation, both doses resulted in a lower percentage of the lung being involved in pneumonia from day 30 onwards (Figure 2B).

**Figure 3:**
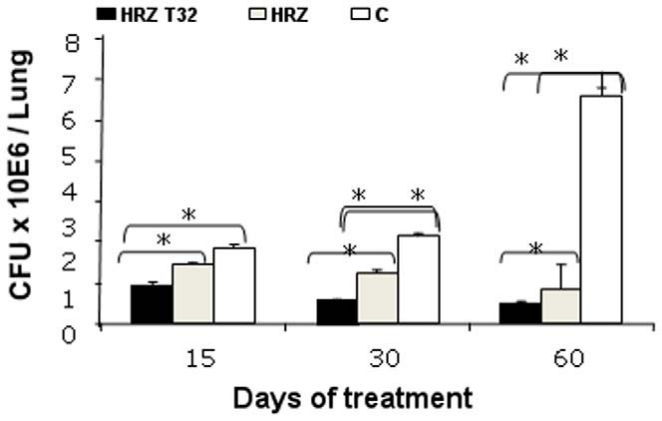
Effect of combined treatment with standard anti-tuberculosis treatment and thioridazine on lung bacillary load in mice infected with *M. tuberculosis* H37Rv. Animals were treated from day 60 with conventional chemotherapy alone (isoniazid [H], rifampicin [R] and pyrazinamide [Z], gray bars), or in combination with thioridazine 32 mg daily (black bars). In comparison with untreated control mice (white bars), both treatments produced significant reduction of bacilli loads after 30 and 60 days of treatment, being higher and faster in the combined treatment group. Data are expressed as means ± SD, 8 mice per time point, asterisks represent statistical significance (p<0.05). T32 HRZ, first line anti-tuberculosis treatment with adjunctive thioridazine 32 mg/kg; HRZ, first line treatment only; C, controls.

In the third experiment, mice infected with H37Rv were treated with conventional chemotherapy alone, or in combination with the 32 mg/kg dose schedule of thioridazine. We chose this dose considering a lower degree of toxicity and a nearly equal efficacy in the reduction of CFUs compared to the highest dose (70 mg/kg) in the second experiment. Both groups receiving only standard anti-tuberculosis therapy showed an important decrease in CFUs at every time point. However, the combination of standard anti-tuberculosis therapy and thioridazine significantly accelerated the reduction of CFUs at every time point of the experiment (Figure 3). In fact, this treatment achieved the fastest rate of clearance of bacilli at day 15 (9×10^5^, SD 1×10^5^ vs 1.46×10^6^, SD 2×10^5^, p<0.04), with CFU values being significantly lower than those achieved by application of the sole standard anti-tuberculosis treatment at days 30 (6×10^5^, SD 1×10^4^ vs 1.24×10^6^, SD 1×10^5^, p<0.006) and 60 (5×10^5^, SD 3×10^4^ vs 8×10^5^, SD 6×10^4^, p<0.01).

**Figure 4:**
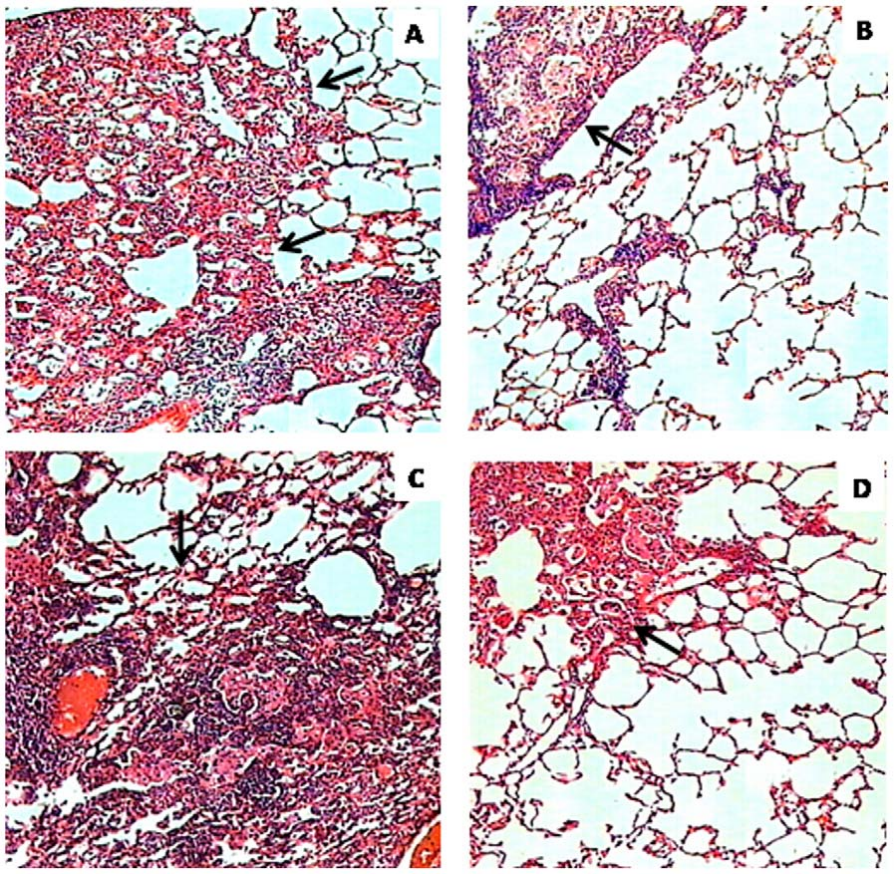
Representative lung histopathology from control- and treated mice after 60 days of thioridazine administration (120 days after infection). (A) Extensive lung consolidation (arrows) is visible in control animals after 120 days of infection by drug-sensible control strain H37Rv. (B) In contrast, less pneumonia (arrow) is seen in the lung of mice treated with thioridazine 32 mg/kg daily by intragastric cannula. (C) Control mice after 120 days of infection with MDR strain show extensive pneumonic areas (arrow). (D) In comparison, less lung consolidation (arrow) is seen in the lung of mice infected by the MDR-TB strain and treated daily during two months with 70 mg/kg of thioridazine (micrographs 200× magnification; hematoxylin/eosin stain).

Thioridazine treatment significantly decreased the extent of lung consolidation. Figure 4 depicts the lung histology after 120 days of infection by *M. tuberculosis* H37Rv (4A–4B) and the MDR-*M. tuberculosis* stain (4C–4D) in untreated mice (Figure 4A and 4C) and in the mice that received thioridazine treatment (Figure 4B and 4D).

## Discussion

Thioridazine treatment results in significant reductions in the number of bacilli in the lung as well as in the percentage of the lungs affected both in susceptible and multidrug-resistant TB in the Balb/c mouse model. The effect of thioridazine was achieved both when the drug was used solely and when applied within a first line antituberculosis regimen. The Balb/c tuberculosis model has been used extensively to test different forms of therapy [12]–[14], confirming that it is highly suitable to explore the efficiency of new drugs or immunotherapy; this is the first study to explore efficacy of a novel drug in MDR-TB infection. The model is based on the airway route of infection which is the most common pathway of infection in humans; intratracheal infection ensures equal distribution over both lungs and accurate control of the infecting dose. The highest rate of bacterial multiplication in the lung correlates with the extent of tissue damage (pneumonia), in coexistence with a bias to Th2 pattern, decline of Th1 response and death of infected animals [9], [14]. We started treatment two months post-infection, a timepoint at which infected animals suffer from progressive tuberculosis with high number of viable bacilli and lung consolidation and mount an increasingly Th2-driven response, to mimic the situation at initial presentation of patients in high-burden, low-income settings. This is different from a previous study by Martins et al. [15] which used a 16 mg/kg dose of thioridazine and started treatment 30 days after intraperitoneal infection of Balb/c mice with 10^6^ CFU; in that study, bacterial loads in the lungs of the mice reduced by 7 logs after 310 days of treatment, but no complete sterilization was achieved. Incomplete sterilisation in that study may have resulted from the use of peritoneal delivery, causing the colonisation of liver and spleen tissues and providing subsequent opportunities for re-infection of the lung. High concentrations of thioridazine are needed for complete inhibition of replication in an aqueous milieu; presumably, mycobacteria in transit via the lymphatic or vascular channels will not be significantly affected by the presence of the compound.

Owing to the high infecting dose with airway delivery, the use of thioridazine monotherapy and, in experiment three, low doses of isoniazid (10 mg/kg) and pyrazinamide (30 mg/kg), the reduction in bacillary load was slower than in previous studies [15], [16]; in a recent study, 6 months of first line treatment with higher isoniazid (25 mg/kg) and pyrazinamide (150 mg/kg) doses were needed to achieve sterilisation, after infection with 1.0×10^5^ viable bacteria [16], as opposed to the 2.5×10^5^ viable bacteria used in the current study.

Thioridazine is now out of patent and its resulting low cost is advantageous in areas where the cost of second-line drugs for MDR-TB bars their use. In areas where second line drugs are available, adding thioridazine to an optimized background regimen may improve therapy outcome, which, to date, has been disappointing [17], [18]. In fact, small-scale studies of the “compassionate use” of thioridazine in XDR-TB patients who failed to respond to any antibiotic regimen have already shown encouraging results [17]. The additional efficacy of thioridazine within an optimized background regimen should be subject of future mouse model studies and thereafter randomized controlled trials.

Importantly, thioridazine is lethal to dormant *M. tuberculosis* in both non-replicating persistent stage 1 and 2 cultures. In the non-replicating persistent stage 1, this is the result of the activity of thioridazine against bacterial respiration. In stage 2, the actual pathway of action remains unknown [19]. As most common anti-tuberculosis drugs are active against actively replicating bacilli only, these dormant bacilli, or persisters, are considered the main reasons for late relapses. Its activity against persisters implies that addition of thioridazine to primary treatment regimens in pan-susceptible TB may prevent relapses and allow shortening of treatment. The synergistic effect of thioridazine when combined with first-line TB treatment (Figure 3), which was previously observed in vitro [20], may be an added advantage in the quest to shorten treatment of pan-susceptible TB. On the other hand, the activity of thioridazine, measured by bacillary load reduction, seems at its maximum in the first month of treatment, to plateau in the second month (Figures 1, 2 and 3). This phenomenon has also been noted in a previous mouse model study, where after 4 months of treatment, activity increased again [15]. This difference in bactericidal activity over time seems to suggest that different cellular functions are targeted at different times or that the availability of a single important target differs over time. Proteomics studies involving the currently known target of thioridazine should address this issue, which may be of critical importance for the inclusion of thioridazine in TB treatment regimens.

Acquisition of thioridazine resistance is a less likely explanation of the decrease in bactericidal activity over time. Thioridazine has multiple mechanisms of action, including interference with cell wall integrity, the binding to calcium transport membranes and inhibition of adherence of calcium, which may be lethal for cells [2], [19], [21]. Therefore, the occurrence of mutations in the genome of *M. tuberculosis* inducing resistance in bacterial (sub)populations is not likely. Recently, the sigma factor network has been described as a protective mechanism for thioridazine-induced cell wall damage of *M. tuberculosis* [21]. Prolonged exposure to thioridazine can lead to partial inactivation of macrophages [22]; whether this affects the immune response against mycobacteria or counteracts the T-cell mediated immunomodulatory effects of thioridazine [8] and partially explains the plateau in CFU counts should be subject of future experiments.

The in vitro minimum inhibitory concentrations (MIC) for pan-susceptible, multidrug- and extensively drug-resistant *M. tuberculosis* isolates are uniform at 4 µg/ml [7]. In humans, thioridazine plasma concentrations up to 4 µg/ml are unachievable; 0.5 µg/ml is the acceptable maximum [23]. Fortunately, *M. tuberculosis* bacteria residing in macrophages are susceptible to thioridazine even at 0.1 µg/ml, since macrophages concentrate thioridazine in the vacuoles where *M. tuberculosis* resides [2], [6]. Lung tissue can efficiently concentrate the phenothiazines [24] and this may explain that the anti-tuberculosis activity in vivo is higher than expected on basis of in vitro MICs. In our murine model, the concentrations of 32 and 70 mg/kg resulted in significant reductions of the number of bacilli and the pathology in the lung. Future studies should investigate the pharmacokinetics of thioridazine in mice and compare these with human data to establish whether the doses chosen in our present study are comparable to maximum dose sizes used in patients.

Interestingly, the MDR *M. tuberculosis* strain used in this study proved less virulent than *M. tuberculosis* H37Rv, based on the lower CFU counts over time in the control groups (Figure 1 and 2). Based on the activity of thioridazine against *M. tuberculosis* H37Rv (Figure 1) and the fact that its efficacy is unrelated to strain type or levels of first- or second-line drug resistance [7], [23], it is likely that its activity would have been equal if a strain of higher virulence had been used. Still, this should be addressed in future studies.

Cardiotoxicity is a concern for all phenothiazines, including thioridazine: in patients managed with thioridazine there are almost twice as many sudden deaths due to cardiac failure as in the general population. Rarely, thioridazine may induce episodes of torsade-de-pointes resulting in sudden death; there are 10 to 15 such events in 10,000 person-years of observation [25]. This issue is complicated by the fact that other drugs involved in treatment of MDR-TB, notably moxifloxacin and to a lesser extent linezolid, have been shown to induce QT-interval prolongation and episodes of torsade-de-pointes [26], [27]. Their combined use with thioridazine may further increase the cardiotoxicity.

In summary, thioridazine shows significant activity against drug-susceptible as well as multidrug-resistant *M. tuberculosis* in a Balb/c mouse model. The phenothiazines and their targets represent interesting novel opportunities in the search for active compounds for treatment of MDR-, or XDR-TB. Its activity against multi- and extensively drug-resistant isolates, low price, good availability, encouraging results in compassionate use and lack of cross-resistance with existing anti-tuberculosis drugs make thioridazine a compound that warrants further investigation. Tolerability of thioridazine in humans is generally good, although cardiotoxicity requires monitoring. With its well-established tolerability and its activity now proven both in vitro and in vivo, thioridazine should continue into the standard work-up of a novel compound with known safety profile: a phase I dose-finding study in TB patients should be done to assess the safety, tolerability, early bactericidal activity and sterilizing capacity; consecutively, a phase II study to proof the concept, followed by a larger phase III study comparing efficacy of an optimized background therapy with and without thioridazine in MDR-TB patients should be done. Simultaneously, more active and less toxic thioridazine derivates should be sought.
